# Lung Protective Ventilation Induces Immunotolerance and Nitric Oxide Metabolites in Porcine Experimental Postoperative Sepsis

**DOI:** 10.1371/journal.pone.0083182

**Published:** 2013-12-12

**Authors:** Jesper Sperber, Miklós Lipcsey, Anders Larsson, Anders Larsson, Jan Sjölin, Markus Castegren

**Affiliations:** 1 Centre for Clinical Research Sörmland, Uppsala University, Uppsala, Sweden; 2 Department of Medical Sciences, Infectious Diseases, Uppsala University, Uppsala, Sweden; 3 Department of Surgical Sciences, Anaesthesiology and Intensive Care, Uppsala University, Uppsala, Sweden; 4 Department of Medical Sciences, Biochemical Structure and Function, Uppsala University, Uppsala, Sweden; University of Cincinnati, United States of America

## Abstract

Low tidal volume ventilation is beneficial in patients with severe pulmonary dysfunction and would, in theory, reduce postoperative complications if implemented during routine surgery. The study aimed to investigate whether low tidal volume ventilation and high positive end-expiratory pressure (PEEP) in a large animal model of postoperative sepsis would attenuate the systemic inflammatory response and organ dysfunction. Thirty healthy pigs were randomized to three groups: Group Prot-7h, i.e. protective ventilation for 7 h, was ventilated with a tidal volume of 6 mL x kg^-1^ for 7 h; group Prot-5h, i.e. protective ventilation for 5 h, was ventilated with a tidal volume of 10 mL x kg^-1^ for 2 h, after which the group was ventilated with a tidal volume of 6 mL x kg^-1^; and a control group that was ventilated with a tidal volume of 10 mL x kg^-1^ for 7 h. In groups Prot-7h and Prot-5h PEEP was 5 cmH_2_O for 2 h and 10 cmH_2_O for 5 h. In the control group PEEP was 5 cmH_2_O for the entire experiment. After surgery for 2 h, postoperative sepsis was simulated with an endotoxin infusion for 5 h. Low tidal volume ventilation combined with higher PEEP led to lower levels of interleukin 6 and 10 in plasma, higher PaO_2_/FiO2, better preserved functional residual capacity and lower plasma troponin I as compared with animals ventilated with a medium high tidal volume and lower PEEP. The beneficial effects of protective ventilation were seen despite greater reductions in cardiac index and oxygen delivery index. In the immediate postoperative phase low V_T_ ventilation with higher PEEP was associated with reduced *ex vivo* plasma capacity to produce TNF-α upon endotoxin stimulation and higher nitrite levels in urine. These findings might represent mechanistic explanations for the attenuation of systemic inflammation and inflammatory-induced organ dysfunction.

## Introduction

Ever since the polio epidemic, mechanical ventilation has been of indisputable value for the survival of many patients with acute respiratory failure [1]. Lung protective ventilation, with low tidal volume (V_T_) ventilation and positive end expiratory pressure (PEEP) titration based on inspiratory oxygen fraction (FiO_2_), has been shown to reduce mortality and morbidity in patients with acute respiratory distress syndrome (ARDS) [2]. Further, in intensive-care treated patients without acute lung injury (ALI) higher V_T_ has been linked to sustained levels of cytokines and to the development of ALI [3]. 

The main effect of protective ventilation is to reduce ventilation-induced lung injury and subsequent spread of inflammation to the systemic compartment, which could reduce the risk of multiple organ failure (MOF) [4]. The effect of ventilator regimes on non-pulmonary organ dysfunction has been investigated in a few animal studies. Brégeon et al., for instance, showed that modes of ventilation that are safe under normal conditions become harmful in the event of systemic inflammation [5]. Imai et al. reported that an injurious ventilator strategy might lead to end-organ epithelial cell apoptosis and organ dysfunction [6] and O'Mahony et al. demonstrated that mechanical ventilation together with endotoxin-enhanced pulmonary inflammation promoted liver and kidney injury [7]. Protective ventilation has been studied in patients during different surgical procedures, however with inconclusive results [8–10]. A retrospective study [11] found that high V_T_ ventilation was associated with a higher occurrence of ARDS and higher mortality in patients with a need for prolonged ventilatory support after surgery. 

Ventilation with low V_T_ and higher PEEP is not the standard for mechanical ventilation on healthy patients during routine surgery. Because of the beneficial outcomes from protective ventilation in injured lungs, it has been proposed by Schultz et al. that low V_T_ ventilation combined with higher PEEP should be used in patients with risk of developing postoperative lung injury [12]. Using the clinical recommendations stated by Shultz et al. and a definition of protective ventilation as V_T_ 6 mL x kg^-1^ and PEEP of 10 cm H_2_O, we hypothesized that protective ventilation might attenuate inflammatory responses in patients undergoing surgery who, due to complications, e.g. anastomotic dehiscence or bowel ischemia, have contracted intraoperative or early postoperative sepsis. This hypothesis was tested in a porcine model. 

The primary aim of this study was to investigate whether low V_T_ 6 mL x kg^-1^ and high PEEP 10 cmH_2_O, in comparison with medium high VT 10 mL x kg^-1^ and low PEEP 5 cmH_2_O, results in an attenuated systemic inflammatory response as measured by plasma levels of tumor necrosis factor α (TNF-α), interleukin 6 and 10 (IL-6 and IL-10). Secondary aims were to study the effect of low V_T_ ventilation and high PEEP on *ex vivo* endotoxin stimulated TNF-α plasma levels, urinary nitrite levels, pulmonary function and inflammatory-induced organ dysfunction and injury. 

An intermediate protective ventilation group, with low V_T_ 6 mL x kg^-1^ and high PEEP 10 cmH_2_O only during the postoperative phase, was added to study possible effects of preventive protective ventilation prior to the postoperative inflammatory stimulus. 

## Materials and Methods

### Ethics statement

The study, approved by the Animal Ethics Board (Uppsala djurförsöksetiska nämnd, permit no. C250/11) in Uppsala, Sweden, included 30 apparently healthy pigs of both sexes with a weight of 25.8±1.5 kg (mean±SD). The pigs were between 9 and 12 weeks old and sexually immature. Water and food access was *ad libitum* until 1 h before the experiment. The pigs were handled in accordance with the animal experimentation guidelines of the Animal Ethics Board in Uppsala, Sweden. Surgery was performed under balanced general anesthesia and all efforts were made to minimize suffering. Humane endpoint during the experiment was signs of pain that were treated with morphine and deepened anesthesia. At the experimental endpoint the animals were sacrificed by way of potassium chloride injection and disconnection from mechanical ventilation.

### Anesthesia and Surgical Procedure

All animals were given 50 mg xylazine intramuscularly immediately before transport to the research facility. General anesthesia was induced by injecting a combination of tiletamine 3 mg x kg^-1^, zolazepam 3 mg x kg^-1^, xylazine 2.2 mg x kg^-1^ and atropine 0.04 mg x kg^-1^ intramuscularly. An intravenous (i.v.) bolus dose of morphine 20 mg and ketamine 100 mg was given before securing the airway with a tracheostomy. The animals were thereafter mechanically ventilated throughout the experiment (Servo 900C or Servo i, Siemens Elema, Stockholm, Sweden). The time of the start of mechanical ventilation was denoted -2 h.

The anesthesia was given as a continuous i.v. infusion of sodium pentobarbital 8 mg x kg^-1^ x h^-1^, morphine 0.26 mg x kg^-1^ x h^-1^ and pancuronium bromide 0.48 mg x kg^-1^ x h^-1^ dissolved in 2.5% glucose solution. Saline 7 mL x kg^-1^ x h^-1^ 0.9% sodium chloride solution was administered i.v., resulting in a total fluid administration rate of 15 mL x kg^-1^ x h^-1^. 

A branch of the right carotid artery was catheterized with a 5F arterial catheter, after which the right external jugular vein was catheterized with a central venous catheter and a 7F Swan-Ganz catheter, the latter placed in the pulmonary artery and advanced with the balloon inflated to facilitate pulmonary arterial wedge pressure. To monitor diuresis a cystostomia catheter was inserted in the bladder through a small laparotomy. A 5F arterial catheter was placed in the left internal jugular vein and advanced 5 cm cranially to approximate placement in the jugular bulb. The left external jugular vein was catheterized with an introducer and a 7F Swan-Ganz catheter advanced under fluoroscopy to enter the hepatic vein. A 20 cm long skin incision was placed below the left costal margin, after which the muscle layers, abdominal fascia and peritoneum were penetrated by blunt dissection. The splenic hilus was identified and the splenic vein catheterized with a 5F arterial catheter, which was advanced 15 cm to reach the portal vein. To confirm correct placement of the catheters in the hepatic and portal veins 5 mL of iohexol contrast medium (Omnipaque™, GE Healthcare AB, Stockholm, Sweden) were injected in the catheters under fluoroscopy.

After completion of the preparations, the animals were given a fluid bolus of Ringer´s acetate solution, 20 mL x kg^-1^ for 10 min, followed by a 30-min stabilization period before the start of the protocol. After the stabilization period, at 1 h 50 min after the start of mechanical ventilation, 0 h values and blood samples were collected, after which the abdominal fascia and skin were closed. An alveolar recruitment maneuver was performed by a stepwise increase in PEEP until an inspiratory plateau pressure of 30 cm H_2_O was reached and inspiratory pressure kept constant for 10 sec. Functional residual capacity (FRC) was measured with the sulfur hexafluoride (SF_6_) inert gas method [13].

### Protocol

**Figure 1:**
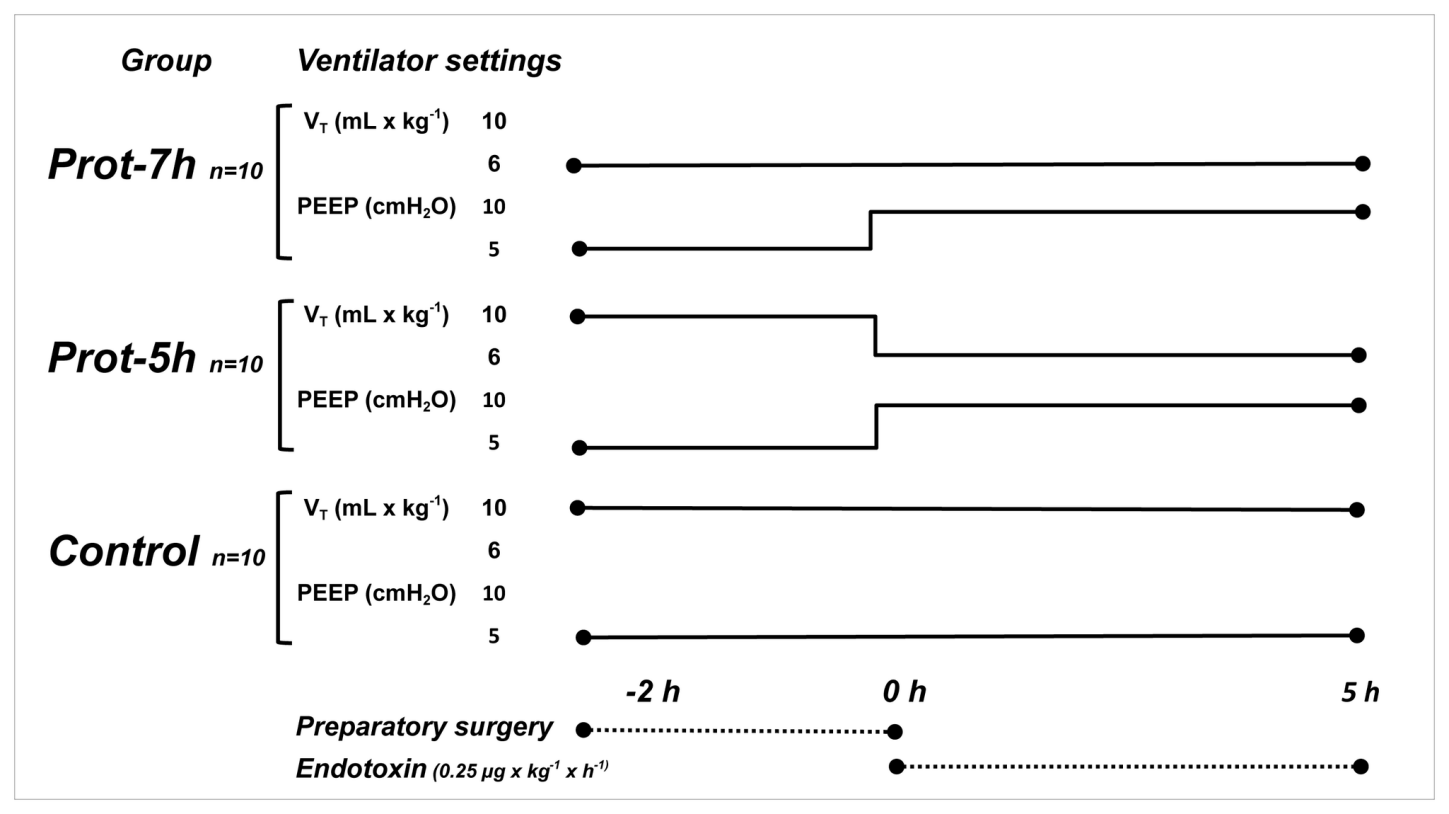
Overview of the experimental design. All groups are n=10. Group Prot-7h was ventilated with low V_T_ 6 mL x kg^-1^ for the entire experiment. Group Prot-5h was ventilated with medium high V_T_ 10 mL x kg^-1^ for 2 h and low V_T_ 6 mL x kg^-1^ during the last 5 h of the experiment. Group Control was ventilated with medium high V_T_ 10 mL x kg^-1^ for the entire experiment. PEEP was 5 cmH_2_O for all groups during the first 2 h and in the control group for the whole experiment. In groups Prot-7h and Prot-5h PEEP was 10 cmH_2_O for the last 5 h. Following preparatory surgery for 2 h, an intravenous endotoxin infusion of 0.25 µg x kg^-1^ x h^-1^ was maintained for the rest of the experiment.

An overview of the study design is given in Figure 1. An i.v. infusion of endotoxin, *E.coli*: 0111:B4 (Sigma Chemical Co., St Louis, MO, USA), was started at 0.25 µg x kg^-1^ x h^-1^ at 0 h. 

The animals were randomized in blocks of 10 to either of three groups: Prot-7h, i.e. protective ventilation for 7 h, (n=10), Prot-5h, i.e. protective ventilation for 5 h, (n=10) and Control, i.e a control group (n=10). Because the experiment aimed to investigate the relative impact of ventilation in experimental postoperative sepsis, no sham animals were included in the experiment. The animals in the Prot-7h group were ventilated with low V_T_, 6 mL x kg^-1^, for the entire experiment. The Prot-5h group was ventilated with a V_T_ of 10 mL x kg^-1^ during experimental surgery, i.e. between -2 h to 0 h, after which V_T_ was adjusted to 6 mL x kg^-1^ for the remaining 5 h. The animals in group Control was ventilated with a V_T_ of 10 mL x kg^-1^ during the entire experiment. All groups were ventilated with a PEEP of 5 cm H_2_0 between -2 h and 0 h. After baseline and during the remaining 5 h of the experiment, groups Prot-7h and Prot-5h were ventilated with a PEEP of 10 cm H_2_0, whereas the control group continued with a PEEP of 5 cm H_2_0. The initial respiratory rate (RR) was 25 x min^-^1 for the groups with V_T_ 10 mL x kg^-1^ and 35 for the group with V_T_ 6 mL x kg^-1^. At -2 h, 0 h and thereafter hourly, the respiration was adjusted to result in arterial carbon dioxide tension (PaCO_2_) between 5.0 - 5.5 kPa. The mode of ventilation was volume controlled with an inspiratory:expiratory (I:E) ratio of 1:2. At 1 h, cefuroxime 20 mg x kg^-1^ was given as a slow injection to prevent bacterial contamination of the model. 

### Interventions

To treat the animals according to intensive care principles a goal-directed intervention protocol was used. This protocol has been used in previous studies [14,15]. Inspired oxygen fraction (FiO_2_) was initially 0.3. Adjustments were made in 0.1 increments of FiO_2_ at an arterial oxygen tension (PaO_2_) <12 kPa and decrements of 0.05 at PaO_2_>18 kPa. PaCO_2_ was kept at values between 5.0 and 5.5 kPa by adjusting RR by increments/decrements of 10%. 

Within 90 minutes from the start of the endotoxin infusion, epinephrine 0.1 mg i.v. was given, maximum twice, if mean arterial pressure (MAP) approximated mean pulmonary arterial pressure (MPAP). Elevation of MPAP is an anticipated response with endotoxin infusion in swine [16,17]. If MAP equaled MPAP after 90 minutes, norepinephrine infusion 20 µg x mL^-1^ i.v. was started with 1 mL bolus and an initial rate of 5 mL x h^-1^. The procedure was repeated with doubling of the infusion rate if MAP relapsed to equaling MPAP. After 90 minutes from the start of the endotoxin infusion, isolated MAP values <50 mmHg were treated with a bolus of Ringer´s Acetate of 10 mL x kg^-1^, maximum 15 mL x kg^-1^ x h^-1^, in addition to the basal fluid protocol. 

### Measurements

MAP, MPAP and central venous pressure (CVP) were monitored continuously. Cardiac output was measured hourly by the thermodilution method. Pulmonary capillary wedge pressure (PCWP) was measured hourly as were proximal airway pressure values, respiratory volumes and urine output. Cardiac index (CI), stroke volume index (SVI), systemic vascular resistance index (SVRI), left ventricular stroke work index (LVSWI), oxygen delivery index (DO_2_I) and static pulmonary compliance were calculated by conventional formulas [18]. FRC was calculated using the SF_6_ inert gas method [13].

Blood samples were drawn from the artery at -2, 0, 1, 3 and 5 h to determine inflammatory cytokines and troponin I. Blood samples were also obtained from the hepatic vein at 0, 1, 3 and 5 h for determination of creatinine and alanine amino transaminase (ALT). The samples were centrifuged to retain plasma, which was frozen at -18°C for later analysis. 

At 0 and 2 h, 1.9 mL of arterial blood were sampled for *ex vivo* endotoxin stimulation. Immediately after the sampling, 0.1 mL of 200 ng x mL^-1^ endotoxin, in the form of purified lipopolysaccharide (LPS) from *E.coli*: 0111:B4 (Sigma Chemical Co., St Louis, MO, USA), were added to each blood sample, resulting in a whole blood concentration of 10 ng endotoxin x mL^-1^. Following incubation at 39°C for 3 h, the blood samples were centrifuged and the supernatants transferred to plasma tubes and stored at -18° until analysis of *ex vivo* endotoxin-stimulated levels of TNF-α.

Arterial, jugular bulb (SjvO_2_), portal vein (SpvO_2_), hepatic vein (ShvO_2_) and mixed venous oxygen saturation (SvO_2_), as well as base excess (BE) were analyzed hourly. Arterial lactate levels were analyzed at 0, 1, 3 and 5 h. Portal lactate levels were analyzed at 0, 1 and 5 h (ABL 5 and Hemoximeter, Radiometer, Brønhøj, Denmark). Blood leukocytes and platelets were analyzed on a CELL-DYN 4000 (Abbott Laboratories, Abbott Park, IL, USA). Analyses of creatinine, ALT and troponin I were performed on an Architect Ci8200 analyzer (Abbott Laboratories, Abbott Park, IL, USA). 

Commercial porcine-specific sandwich enzyme-linked immunosorbent assay (ELISA) was used for the determination of TNF-α, IL-6 and IL-10 in plasma (DY690B (TNF-α) and DY686 (IL-6), R&D Systems, Minneapolis, MN, USA and KSC0102 (IL-10), Invitrogen, Camarillo, CA, USA). The ELISAs had an intra-assay coefficient of variation (CV) of less than 5% and a total CV of less than 10%. 

After enzymatic conversion of nitrate to nitrite by nitrate reductase, total nitrite concentration in urine at 0, 2 and 4 h was measured using the Parameter™ assay (SKGE001, R&D Systems, Minneapolis, MN, USA). The urine samples were diluted 1:5 before the assay according to the recommendations of the manufacturer.

### Endpoints, calculations and statistics

The primary endpoint of this experiment was to detect differences in TNF-α, IL-6 and IL-10 concentrations over time during the entire experiment. Therefore, only animals that survived the entire experiment were included in the experiment. If an animal died before the experimental endpoint, the animal was excluded and replaced. The primary endpoint variables TNF-α, IL-6 and IL-10 concentrations were log-normally distributed and therefore these values were logarithmically transformed for the statistical analyses. 

Baseline differences for variables approximating a normal distribution were analyzed with multiple analysis of variance (MANOVA) comparing differences between all three groups at baseline, whereas baseline differences for non-normally distributed variables were analyzed with Kruskall-Wallis test at baseline.

The main statistical analysis for the primary endpoint variables was MANOVA for repeated measures, analyzing the group effect between all three groups during the entire experimental period and not at individual time points. The time and group by time effects in MANOVA for repeated measures were not analyzed. The same strategy was applied to the normally distributed secondary outcome variables. Only if the MANOVA for repeated measures yielded a significant difference between the groups, *post hoc* analyses were performed using ANOVA for repeated measures comparing differences between the individual groups during the entire experiment. Secondary outcome variables with a normal distribution were indexed to reduce the effect of inter-animal variation. Subsequently variables were expressed as the percentual change in relation to the first measurement, i.e. at -2 h or 0 h depending on first available sample. 

Variables that were non-normally distributed, i.e. Troponin I, FRC, total urinary nitrite and ΔTNF-α, i.e. the difference between TNF-α values after and before *ex vivo* endotoxin stimulation, were analyzed for group differences between individual groups with Mann-Whitney *U* test for each time point from 0 to 5 h. 

A *p-value* <0.05 was considered statistically significant. Data with a normal distribution are presented as mean ± standard deviation (SD); data with a non-normal distribution are presented as median (interquartile range). Statistica™ (Statsoft, Tulsa, OK) was used in the statistical calculations and for the control of relevant assumptions. The statistical design and analyses were reviewed by a senior statistician.

## Results

Two of the animals that were initially randomized to group Prot-5h died before the experimental endpoint and were hence excluded from the experiment and replaced according to the statistical plan. Both animals died in association with the initial pulmonary hypertension seen during porcine endotoxemia [16,17]. 

Epinephrine was given to five animals in the Prot-7h group, six animals in the Prot-5h group and three animals in the control group. Norepinephrine infusion was given to one animal in each group. The results from the animals requiring vasoactive drugs were well within the variation of those from other animals in the respective groups. Fluid boluses between 0 and 5 h were given to 5 animals in the Prot-7h group and 7 animals in the Prot-5h group, with median challenges of 17.5 and 5 mL x kg^-1^ (ranges 0-40 and 0-15), respectively. In the control group only two animals needed fluid boluses. Adjustments of ventilator settings were made in all groups according to protocol. The ventilator settings as well as airway pressures and static pulmonary compliance are listed in Table 1. 

**Table 1:** Ventilator settings, airway pressures and static pulmonary compliance during the experiment.

		**-2 h**	**0 h**	**3 h**	**5 h**
**Tidal volume (mL)**	**Prot-7h**	157±7	159±14	159±12	146±48
	**Prot-5h**	265±17	264±13	161±11	161±11
	**Control**	257±15	261±26	264±26	264±24
**RR (min^-1^)**	**Prot-7h**	43±8.9	45±10	50±11	50±15
	**Prot-5h**	21±4	18±3	45±11	49±11
	**Control**	20±4	20±5	22±3	23±5
**PEEP (cmH_2_O**)	**Prot-7h**	5±0	5±0	10±0	10±1
	**Prot-5h**	5±0	5±0	10±0	10±0
	**Control**	5±0	5±0	5±0	5±0
**Pplat (cmH_2_O)**	**Prot-7h**	15±3	16±4	22±4	22±3
	**Prot-5h**	16±3	17±2	20±4	21±3
	**Control**	17±3	17±3	21±3	22±3
**pCO_2_ (kPa)**	**Prot-7h**	5.8±0.9	5.4±0.7	5.8±0.6	5.5±0.6
	**Prot-5h**	4.3±0.5	5.2±0.5	5.5±0.3	5.3±0.1
	**Control**	5.0±0.5	5.1±0.4	5.4±0.7	5.6±0.6
**Compliance (mL x cmH_2_O** ^**-**1^)	**Prot-7h**	18±5	16±5	15±5	13±6
	**Prot-5h**	30±20	23±5	19±13	17±9
	**Control**	23±6	23±4	17±3	16±3

All groups are n=10. Values are mean ± SD.

RR = respiratory rate, PEEP = positive end expiratory pressure, Pplat = plateau pressure pCO_2_ = arterial CO_2_ tension.

 The blood gas derived variables BE, pH and PaCO_2_ differed between the groups at -2 h. After adjustment of the ventilator settings according to protocol, these differences were corrected and no longer existed at 0 h. No other variables differed between the groups at -2 or 0 h. 

### Inflammatory response

**Figure 2:**
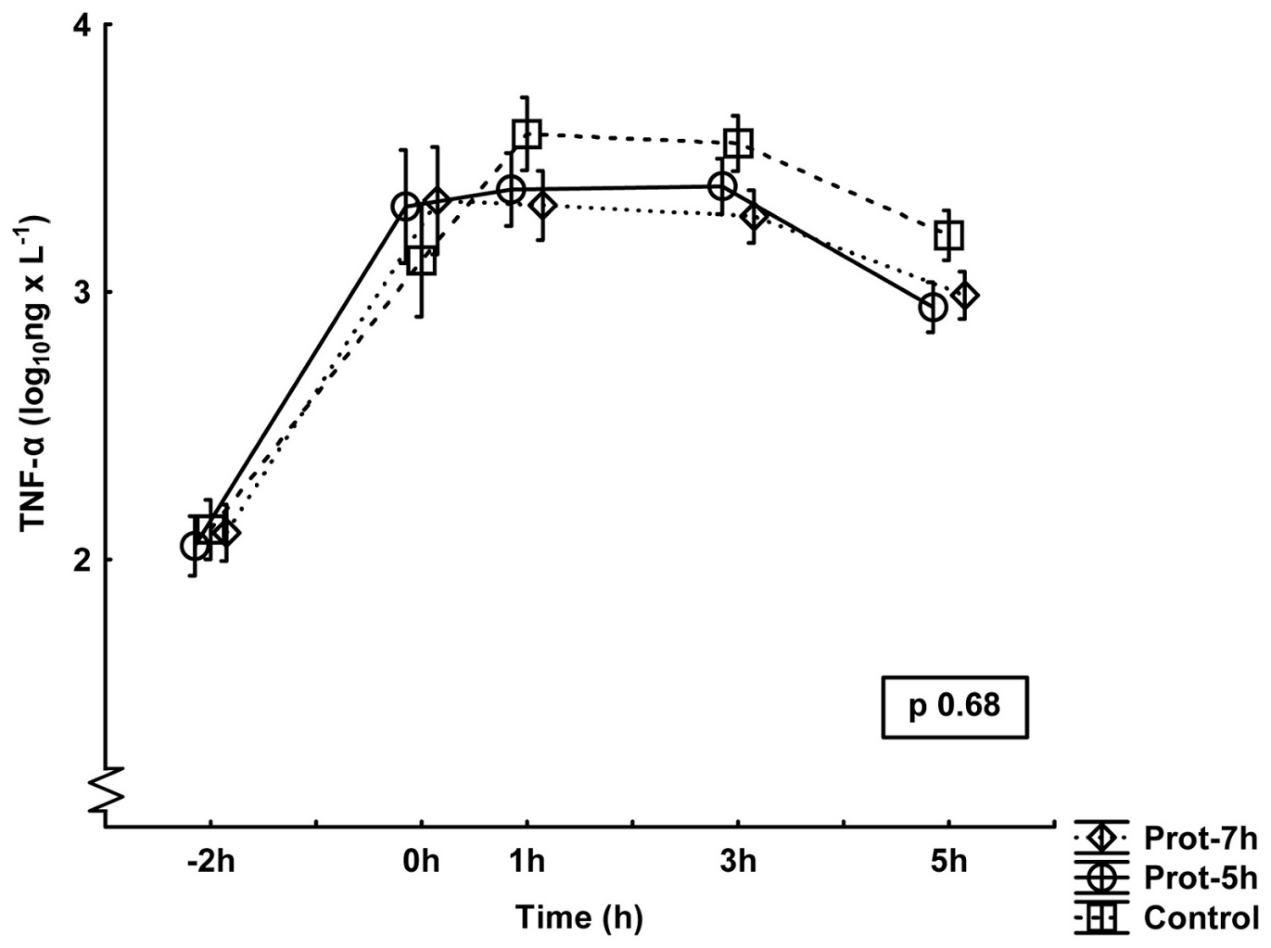
Plasma levels of tumor necrosis factor α (TNF-α) during the experiment. All groups are n=10. The values have been logarithmically transformed. Mean±SE. The p-value is the result of a multiple ANOVA (MANOVA) for repeated measures comparing differences between all three groups during the entire experiment period.

**Figure 3:**
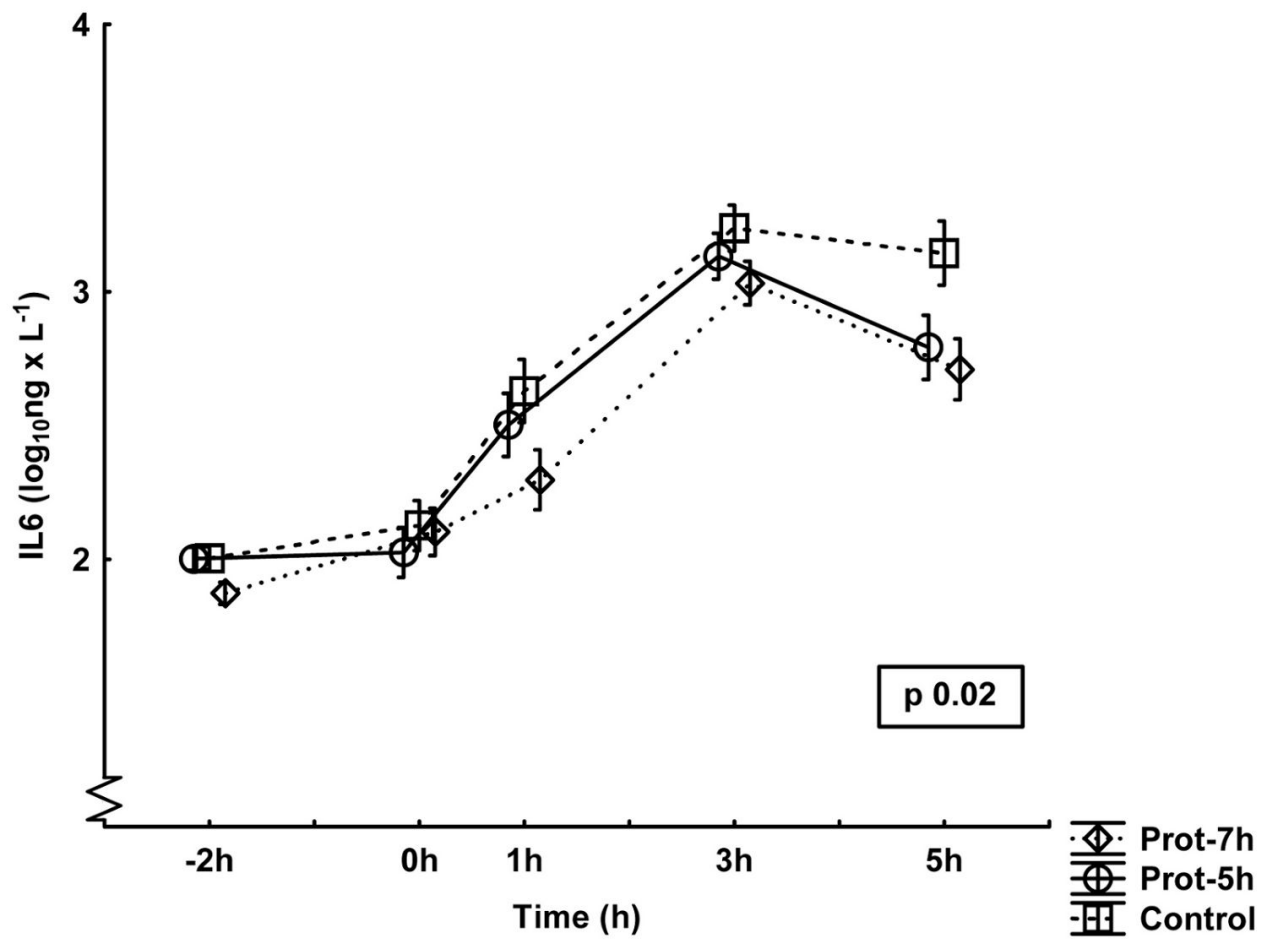
Plasma levels of interleukin 6 (IL-6) during the experiment. All groups are n=10. The values have been logarithmically transformed. Mean±SE. The p-value is the result of a multiple ANOVA (MANOVA) for repeated measures comparing differences between all three groups during the entire experiment period.

**Figure 4:**
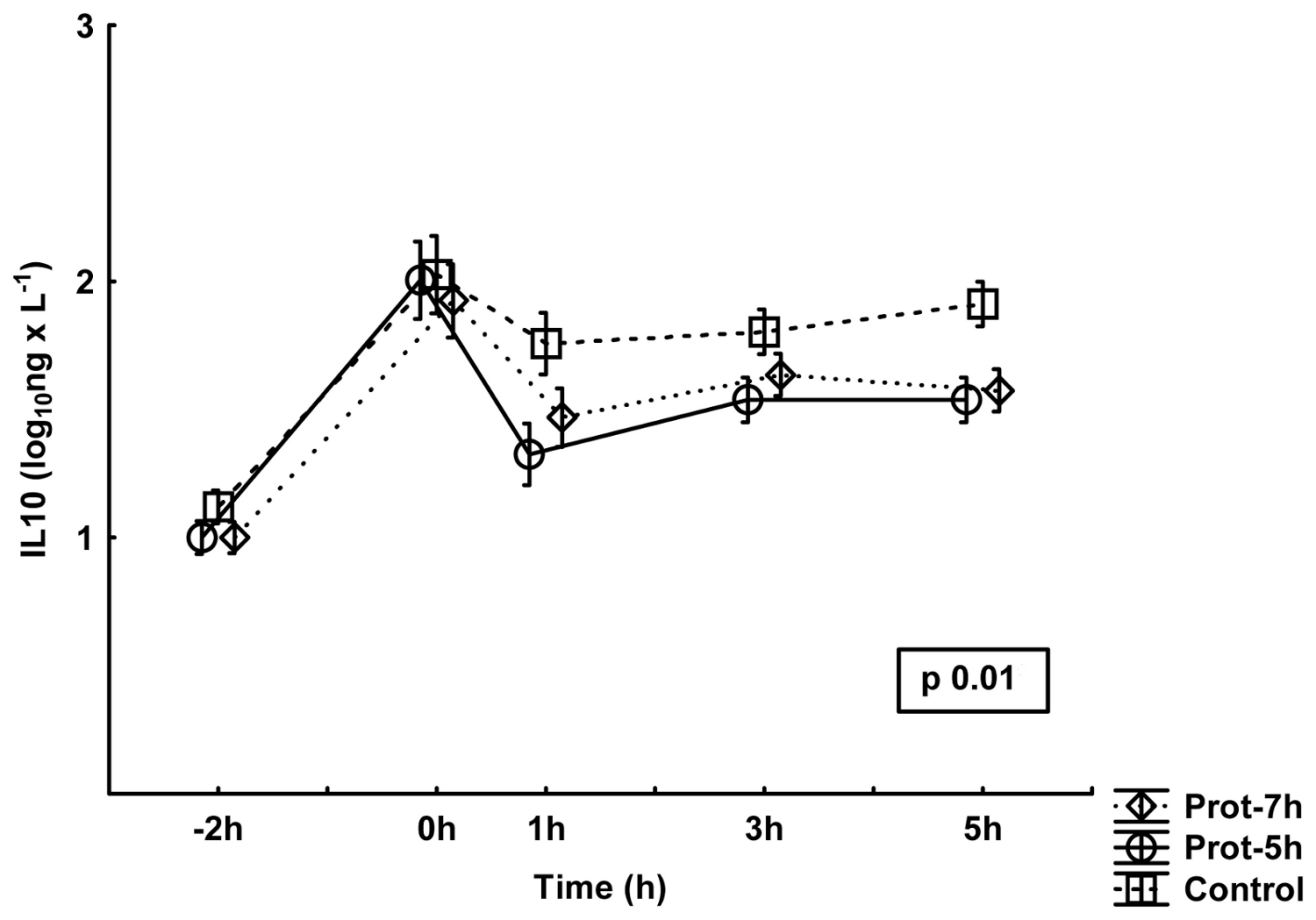
Plasma levels of interleukin 10 (IL-10) during the experiment. All groups are n=10. The values have been logarithmically transformed. Mean±SE. The p-value is the result of a multiple ANOVA (MANOVA) for repeated measures comparing differences between all three groups during the entire experiment period.

The responses in TNF-α, IL-6 and IL-10 are presented in Figures 2,3,4. No differences between groups were noted for the TNF-α values, although the control group showed numerically higher values than the other groups toward the end of the experiment. There were significant differences between groups in IL6 and in IL10. *Post hoc* analysis demonstrated lower IL-6 values in the Prot-7h group than in the control group (p<0.05), with the most marked differences at the end of the experiment. IL-10 values were, in the *post hoc* analyses, lower in the Prot-7h (p<0.05) and Prot-5h (p<0.05) groups than in controls, with the most marked differences at the end of the experiment. 

**Figure 5:**
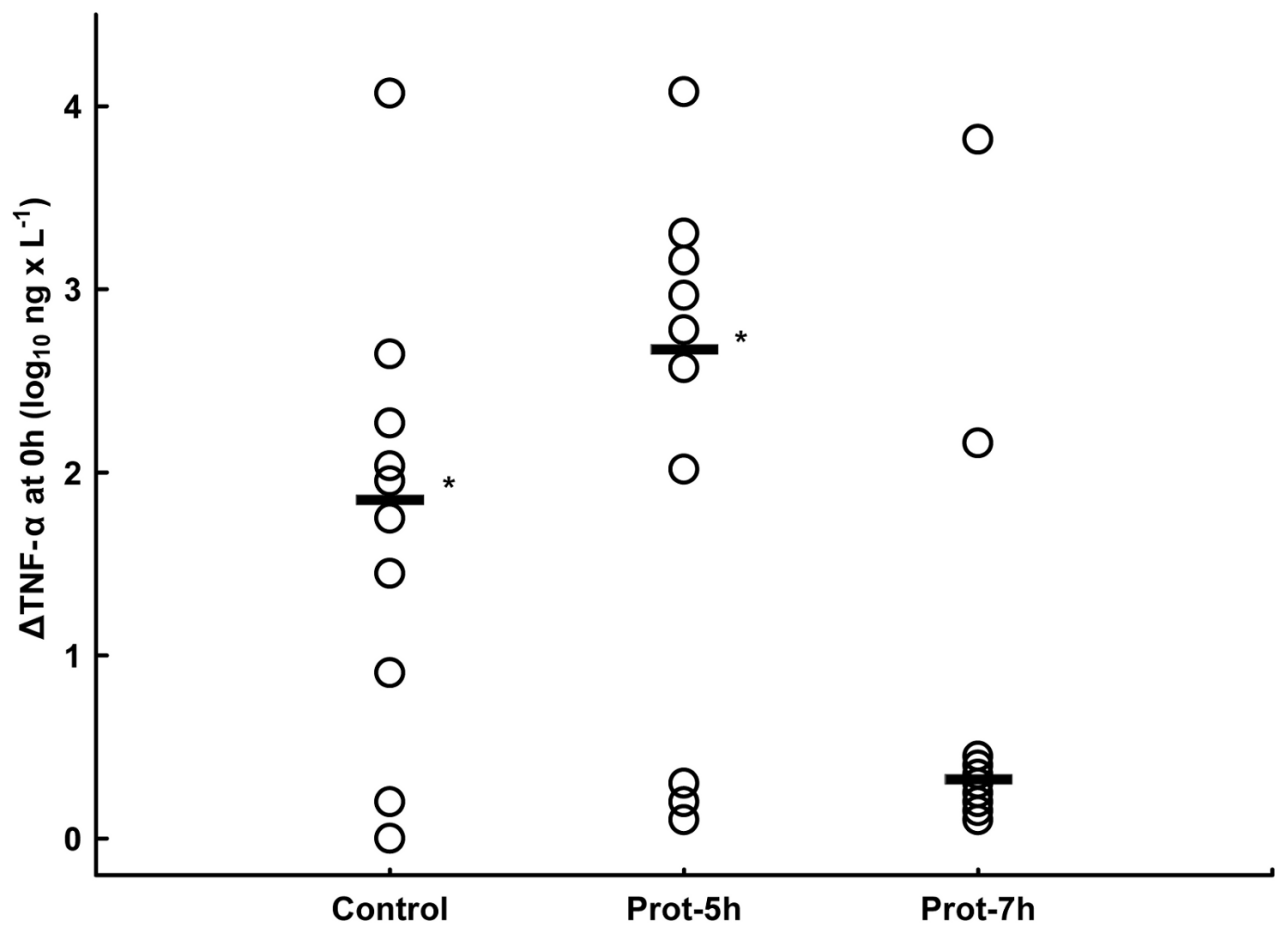
Endotoxin-induced whole blood tumor necrosis factor α production *ex* *vivo*, Δ-TNF-α, in the immediate postoperative period at 0 h. All groups are n=10. The values are given on a logarithmic scale as a scatterplot. The horizontal bar denotes the median. The * denotes a significant difference compared with the Prot-7h group, p<0.05, Mann-Whitney *U* test.

The propensity to produce TNF-α after *ex vivo* endotoxin stimulation at 0 h, ΔTNF-α, was lower in the Prot-7h group, where 8/10 animals showed a completely suppressed cytokine production *ex vivo* compared with the Prot-5h (p<0.05) and control groups (p<0.05) (Figure 5). At 2 h, complete suppression of ΔTNF-α in the Prot-7h group was no longer present and differences between the groups were no longer apparent (data not shown). 

**Figure 6:**
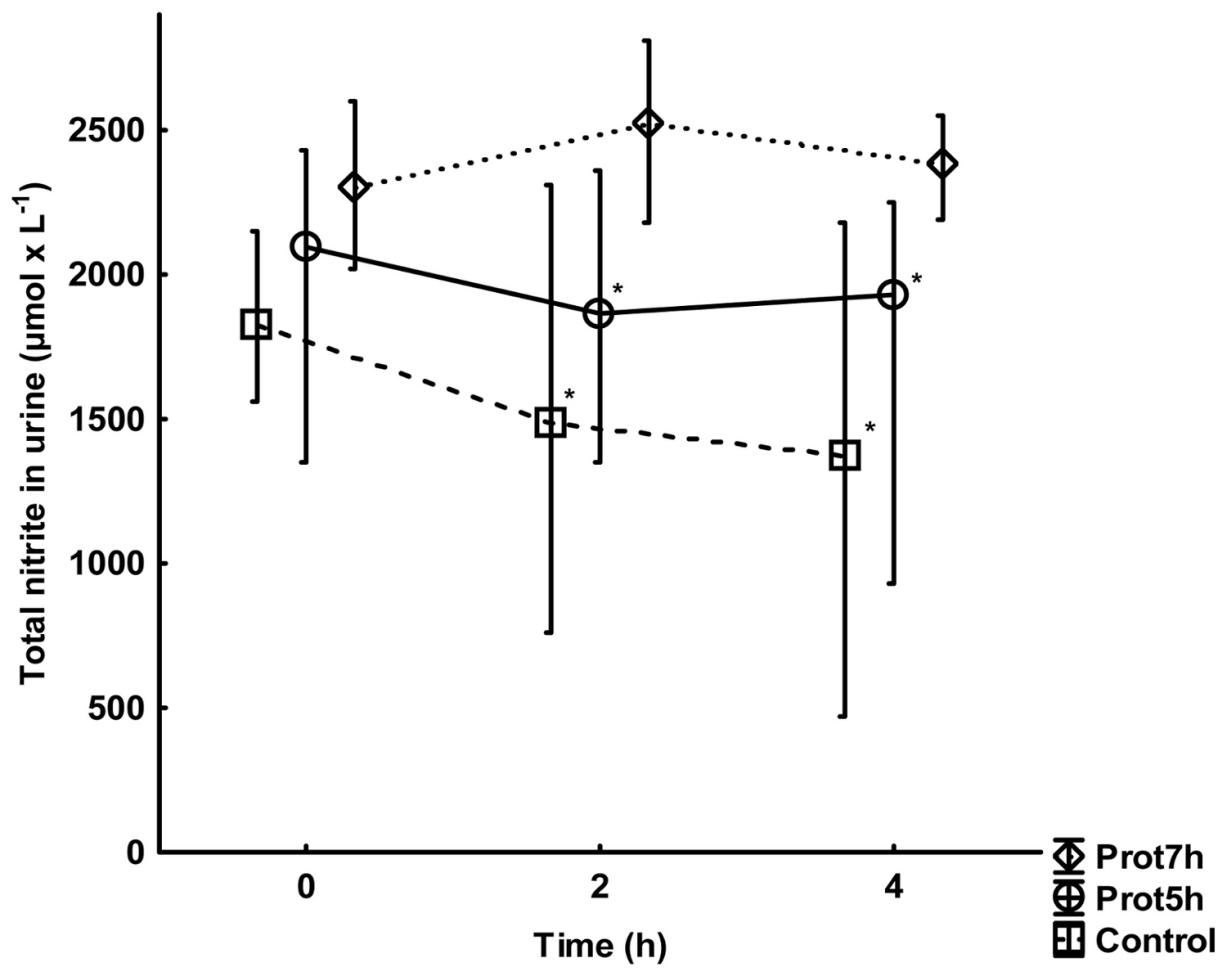
Total nitrite concentration in urine. All groups are n=10. Values are median and interquartile range. The * denotes a significant difference compared with the Prot-7h group, p<0.05, Mann-Whitney *U* test.

Urinary total nitrite was higher at 2 and 4 h in the Prot-7h group than in both the control group (p<0.05 and p<0.05 respectively) and the Prot-5h group (p<0.05 and p<0.05 respectively) (Figure 6). 

Differences could not be established between groups in neutrophil, total leukocyte or platelet counts (Table 2).

**Table 2:** Inflammatory cell counts and pulmonary function variables during the experiment.

		**-2 h**	**0 h**	**3 h**	**5 h**	**p**
**Neutrophils (10^9^x L^-1^**)	**Prot-7h**	8.0±3.0	3.1±2.6(-64±21%)	5.7±8.2(-35±55%)	4.6±5.2(-47±33%)	
	**Prot-5h**	7.7±4.7	3.0±3.5(-66±15%)	5.5±7.5(-40±39%)	4.8±5.7(-45±31%)	**0.35**
	**Control**	10.3±7.6	3.9±4.1(-63±18%)	4.3±4.3(-59±33%)	3.8±2.7(-55±33%)	
**Leukocytes (10^9^x L^-1^**)	**Prot-7h**	18.6±4.0	9.0±3.1(-52±12%)	9.7±8.5(-48±34%)	8.1±5.2(-57±20%)	
	**Prot-5h**	18.4±5.1	9.1±4.1(-52±11%)	9.9±7.7(-50±22%)	8.4±5.8(-46±17%)	**0.44**
	**Control**	20.4±10.0	9.6±5.4(-53±14%)	8.4±5.0(-59±17%)	7.2±3.5(-62±17%)	
**Platelets (10^9^x L^-1^**)	**Prot-7h**	526±192	402±113(-20±8%)	298±128(-41±10%)	272±124(-48±11%)	
	**Prot-5h**	473±100	375±97(-21±8%)	265±85(-45±10%)	244±73(-49±8%)	**0.71**
	**Control**	483±104	389±93(-19±12%)	280±68(-42±9%)	255±93(-48±13%)	
**PaO_2_/FiO_2_ (mmHg**)	**Prot-7h**	460±49	468±58(2±8%)	398±86(-13±16%)	393±201(-15±36%)	
	**Prot-5h**	518±49	488±65(-6±10%)	364±127(-30±22%)	346±108(-33±22%)	**0.03**
	**Control**	457±90	473±70(5±10%)	274±100(-39±20%)	219±88(-50±21%)	
**FRC (mL)**	**Prot-7h**	-	748(537-885)	-	666(515-745)	
	**Prot-5h**	-	740(662-868)	-	722(589-943)	**N/A**
	**Control**	-	642(594-667)	-	472(396-572)	

All groups are n=10. The first obtained value in each variable is presented as absolute, thereafter as absolute and percentage change from the first value, mean ± SD. FRC values are absolute, median and interquartile range. The p-values are results of multiple ANOVA (MANOVA) for repeated measures comparing the percentage changes from baseline between all three groups during the entire experiment period. FRC was analyzed with Mann-Whitney *U* test.

PaO_2_/FiO_2_ = arterial oxygen tension/inspired oxygen fraction, FRC = functional residual capacity, N/A = not applicable.

### Respiration

PaO_2_/FiO_2_ displayed significant differences between groups. *Post hoc* analyses showed that the Prot-7h group had higher values than the control group during the experiment (p<0.05) whereas only a trend toward significance compared to the Prot-5h group (p=0.07) was seen. These differences were most evident towards the end of the experiment (Table 2). 

FRC was higher at 5 h in the Prot-5h group than in the control group (p<0.05), whereas only a trend toward a difference was evident between the Prot-7h and the control groups (p=0.07) (Table 2).

### Circulation

Significant differences between groups were seen in the variables CI, DO_2_I, LVSWI and SVI. In MAP, PCWP, SVRI and CVP (data not shown), no differences between the groups were evident. 

*Post hoc* analyses revealed that the Prot-7h group decreased more in CI, most evident at 5 h, than the control group (p<0.01) (Table 3). A highly significant reduction in DO_2_I was found in the Prot-7h group as compared with the control group (p<0.01). In LVSWI both the Prot-7h and Prot-5h groups showed significantly more reduced values than the control group (p<0.05 and p<0.01). SVI decreased significantly more in group Prot-5h compared to the control group (p<0.01) whereas only a trend towards significance was seen between Prot-7h and the control group (p=0.08). 

**Table 3:** Circulatory variables.

		**-2 h**	**0 h**	**3 h**	**5 h**	**p**
**CI (L x min^-1^ x m^-2^)**	**Prot-7h**	3.5±0.5	2.8±0.6(-20±10%)	2.0±0.4(-43±15%)	2.1±0.6(-39±18%)	
	**Prot-5h**	3.2±0.8	2.7±0.5(-9±25%)	1.6±0.4(-47±17%)	1.8±0.4(-39±21%)	**0.04**
	**Control**	2.9±0.5	2.6±0.5(-8±18%)	2.3±0.4(-20±18%)	2.2±0.5(-23±18%)	
**DO_2_I (mLO_2_ x min^-1^ x m^-2^ )**	**Prot-7h**	434±70	336±71(-23±9%)	239±42(-44±14%)	238±48(-44±13%)	
	**Prot-5h**	415±102	341±66(-14±26%)	217±41(-45±17%)	227±44(-42±20%)	**0.02**
	**Control**	367±76	330±70(-9±20%)	289±57(-19±19%)	270±53(-25±15%)	
**LVSWI (g x m^-2^)**	**Prot-7h**	49±13	33±8(-32±15%)	17±9(-62±23%)	15±7(-68±18%)	
	**Prot-5h**	47±11	33±10(-26±26%)	15±11(-70±16%)	11±2(-75±8%)	**0.005**
	**Control**	38±11	32±8(-10±31%)	21±7(-40±30%)	15±6(-55±29%)	
**SVI (mL x m^-2^)**	**Prot-7h**	35±7	30±5(-14±12%)	19±3(-43±19%)	20±5(-40±22%)	
	**Prot-5h**	34±5	28±7(-16±24%)	16±6(-54±15%)	17±4(-49±15)	**0.01**
	**Control**	28±6	28±4(1±23%)	19±3(-31±19%)	17±4(-35±25)	
**MAP (mmHg)**	**Prot-7h**	110±8	88±10(-20±9%)	77±24(-30±21%)	65±17(-41±13%)	
	**Prot-5h**	107±13	93±11(-12±15%)	75±24(-30±20%)	60±8(-43±9%)	**0.07**
	**Control**	106±13	92±9(-12±12%)	90±19(-14±20%)	71±14(-32±18%)	
**PCWP(mmHg)**	**Prot-7h**	7.1±2.4	7.3±2.8(3±24%)	12.3±3.2(87±77%)	11.5±3.2(73±66%)	
	**Prot-5h**	6.2±1.9	6.0±1.9(0±30%)	10.0±2.2(73±56%)	9.5±1.4(65±50%)	**0.09**
	**Control**	7.4±2.2	8.1±2.5(10±21%)	9.3±1.9(32±35%)	9.4±2.8(30±24%)	
**SVRI (dynes-sec x cm^-5^ x m^-2^)**	**Prot-7h**	2397±252	2424±626(0±21%)	2835±1112(18±43%)	2146±793(-10±31%)	
	**Prot-5h**	2725±788	2639±756(1±30%)	3404±1216(29±40%)	2427±811(-7±29%)	**0.48**
	**Control**	2811±641	2679±651(-4±18%)	2946±537(+8±22%)	2389±641(-14±16%)	

All groups are n=10. The first obtained value in each variable is presented as absolute, thereafter as absolute and percentage change from the first value, mean ± SD. The p-values are results of multiple ANOVA (MANOVA) for repeated measures comparing the percentage changes from baseline between all three groups during the entire experiment period.

CI = cardiac index, DO_2_I = oxygen delivery index, LVSWI = left ventricular stroke work index, SVI = stroke volume index, MAP = mean arterial pressure, PCWP = pulmonary capillary wedge pressure, SVRI = systemic vascular resistance index.

### Hypoperfusion and organ dysfunction

Significant differences were found in SjvO_2_, where *post hoc* analyses showed that group Prot-5h decreased more than the control group (p<0.05), whereas the Prot-7h group and the control group only showed a trend towards a difference (p=0.08) (Table 4). The animals in the control group increased more in troponin I at the end of the experiment compared with the animals in the Prot-5h group (p<0.05). Between the control group and the Prot-7h group only a trend towards a difference was seen (p=0.08) (Table 4).

**Table 4:** Hypoperfusion and organ dysfunction variables.

		**-2h**	**0h**	**3h**	**5h**	**p**
**SjvO_2_(%)**	**Prot7h**	-	74.6±10.8	54.5±18.2(-27±22%)	55.4±16.6(-25±22%)	
	**Prot5h**	-	76.8±11.2	46.4±21.1(-41±22%)	54.1±18.3(-30±21%)	**0.02**
	**Control**	-	72.0±18.7	63.9±17.3(-5±35%)	61.5±20.7(-10±32%)	
**Troponin I(mmol x L^-1^)**	**Prot7h**	**-**	0.10(0.06-0.11)	-	0.14(0.09-0.50)	
	**Prot5h**	**-**	0.09(0.03-0.17)	-	0.19(0.05-0.31)	**N/A**
	**Control**	**-**	0.08(0.04-0.11)	-	0.36(0.17-0.60)	
**pH(-log[H⁺])**	**Prot7h**	7.45±0.06	7.47±0.04	7.41±0.05(-6±7%)	7.43±0.06(-4±7%)	
	**Prot5h**	7.56±0.04	7.48±0.05	7.41±0.05(-7±4%)	7.43±0.03(-5±3%)	**0.77**
	**Control**	7.49±0.04	7.48±0.03	7.44±0.07(-4±7%)	7.42±0.09(-6±8%)	
**Creatinine(mmol x L^-1^**)	**Prot7h**	**-**	88± 17	86±17(0± 13%)	93±27(7± 2%)	
	**Prot5h**	**-**	86± 11	82±8(-4± 9%)	89±10(5± 18%)	**0.94**
	**Control**	**-**	85± 14	83±17(-2± 14%)	91±20(8± 22%)	
**ALT(µkat x L^-1^)**	**Prot7h**	**-**	0.6± 0.3	0.5±0.2(-9± 32%)	0.6±0.2(-5± 29%)	
	**Prot5h**	**-**	0.7± 0.2	0.6±0.2(-9± 25%)	0.5±0.2(-22± 26%)	**0.58**
	**Control**	**-**	0.8± 0.2	0.7±0.2(-14± 18%)	0.7±0.2(-24± 22%)	
**Lactate arterial(mmol x L^-1^)**	**Prot7h**	**-**	2.0±0.4	2.7±0.7(37± 39%)	1.8±0.8(-4.9± 43%)	
	**Prot5h**	**-**	2.4±0.4	3.5±0.7(46± 31%)	2.7±0.6(15± 26%)	**0.97**
	**Control**	**-**	2.2±0.1	2.7±1.0(34± 51%)	2.5±1.0(24± 57%)	
**SpvO_2_(%)**	**Prot7h**	-	72.0±17.7	57.5±11.1(-17±27%)	57.0±10.1(-15±27%)	
	**Prot5h**	-	65.6±17.9	46.1±15(-28±19%)	57.1±18,8(-15±19%)	**0.27**
	**Control**	-	79.6±14.1	60.9±20.0(-25±18%)	53.9±21.6(-35±21%)	
**ShvO_2_(%)**	**Prot7h**	-	35.9±9.6	15.3±7.6(-54±33%)	20.0±12.4(-38±62)	
	**Prot5h**	-	44.7±9.7	14.8±8.1(-64±24%)	18.9±9.9(-58±17%)	**0.12**
	**Control**	-	40.2±11.9	22.0±16.8(-51±28%)	21.6±12.6(-38±55%)	
**Lactate portal vein(mmol x L^-1^)**	**Prot7h**	**-**	2.0±0.4	-	1.7±1.0(-11±56%)	
	**Prot5h**	**-**	2.5±0.4	-	3.1±0.8(23±23%)	**0.06**
	**Control**	**-**	2.1±1.0	-	3.0±1.3(53±70%)	

All groups are n=10. The first obtained value in each variable, except for the logarithmical variable pH, is presented as absolute, thereafter as absolute and percentage change from the first value, mean ± SD. pH is log normal, mean ± SD. Troponin I values are absolute, median and interquartile range. The p-values are results of multiple ANOVA (MANOVA) for repeated measures comparing the percentage changes from baseline between all three groups during the entire experiment period. Troponin I was analyzed with Mann-Whitney *U* test. SjvO_2_ = jugular vein oxygen saturation, ALT = alanine amino transferase, SpvO_2_= portal vein oxygen saturation, ShvO_2_= hepatic vein oxygen saturation, N/A = not applicable.

There were no differences in base excess (data not shown), pH, creatinine, alanine amino transferase (ALT), arterial lactate levels, SpvO_2_ or ShvO_2_ between the groups. Lactate levels in the portal vein showed a trend towards significance (Table 4).

## Discussion

The study demonstrates that low V_T_ ventilation together with higher PEEP, i.e. protective ventilation, in healthy animals under general anesthesia attenuates the systemic inflammatory response during experimental postoperative sepsis. The data thus lend support to the hypothesis of this study as well as to the recommendation to use low V_T_ ventilation together with higher PEEP in patients at high risk of postoperative complications [12]. 

 The attenuation of the inflammatory response was measured as lower plasma levels of IL-6 and IL-10 in the protectively ventilated animals. The lung protective ventilation also led to less pulmonary dysfunction and better preserved FRC after 5 h of endotoxemia. At the end of the experiment, the animals that were ventilated with medium high V_T_ and lower PEEP had decreased more than 50% from baseline in PaO_2_/FiO_2_, leading to values only slightly above the ARDS definition (200 mmHg). The protectively ventilated animals, however, only showed a slight decrease in PaO_2_/FiO_2_ during the experiment, expressing values well above the ALI definition (300 mmHg) at the end of the experiment (Table 2). The animals that were ventilated with low V_T_ and higher PEEP only during the last 5 h, showed an intermediate response to the other groups. However, the effects were not large enough to reach statistical significance compared with the animals that were ventilated with the protective ventilation protocol during the entire experiment. 

The concept of lung biotrauma and subsequent non-pulmonary organ injury has attracted scientific interest as a model of MOF [19–21]. The animals ventilated with a low V_T_ increased less in plasma troponin I than the animals in the control group, indicating a less prominent endotoxin-induced cardiac injury following preventive lung protective ventilation. Lower lactate levels in the portal vein, although not reaching significance, were seen at the end of the experiment in the animals ventilated with the protective protocol (Table 4). The portal lactate levels increased more than the arterial lactate levels during the experiment, indicating that the trend towards lower portal lactate levels in the protectively ventilated animals may be attributed to improved splanchnic circulation. These beneficial results of low V_T_ ventilation on non-pulmonary organ injury are especially noteworthy considering that they were observed despite decreased macro-circulatory variables, e.g. reduced CI and DO_2_I. The greater decrease in macro-circulatory variables seen in the protectively ventilated animals is probably explained by the higher PEEP, leading to higher intrathoracic pressure and subsequent lower venous return [22].

Protective ventilation was associated with reduced SjvO_2_. No differences in splanchnic or hepatic oxygen extraction could be detected in this study, as reflected by the lack of differences between the groups in SpvO_2_ and ShvO_2_. Although the mean SjvO_2_ in all groups decreased during the experiment, the levels approximated the lower normal range in humans [23]. The differences between the groups could be partly attributed to the differences in DO_2_I. 

In a recent publication, using a porcine endotoxemic model, lower endotoxin-stimulated TNF-α production *ex vivo* was associated with a hyperdynamic circulatory state, i.e. lower blood pressure, higher CI and lower SVRI [15]. In the present study the animals preventively ventilated with low V_T_ and higher PEEP, i.e. during experimental surgery, demonstrated lower endotoxin-induced TNF-α production *ex vivo*, i.e. Δ-TNF-α, at the start of endotoxin infusion (Figure 5). In contrast to the other variables, Δ-TNF-α interestingly separates the two protective groups significantly, indicating that the difference in tidal volume and the subsequent difference in respiratory rate, the only differences between the groups at this time, influence the immune system with a rapid response. These differences were no longer present 2 h later, but because the animals at that time had been exposed to a continuous endotoxin infusion for 2 h, the reaction to *ex vivo* endotoxin is difficult to compare with that at 0 h. 

The induction of endotoxin tolerance or immunotolerance is highly complex as recently modeled by Fu et al. [24]. Endotoxin tolerance is normally defined as a reduced endotoxin-induced production of inflammatory cytokines after previous exposure to endotoxin [25]. Endotoxin tolerance has been observed in patients with sepsis, trauma, pancreatitis and in patients surviving cardiac arrest [26]. Moreover, it has been associated with the level of disease in ARDS [27]. The role of inducible nitric oxide synthase (iNOS) in the induction of endotoxin tolerance was elegantly shown by Dias et al. in a murine model in which endotoxin tolerant animals given the specific iNOS antagonist aminoguanidine returned to the native response to endotoxin in a similar manner to iNOS knockout mice [28]. Vobruba et al. published higher nitrite/nitrate and iNOS levels in bronchoalveolar lavage from pigs ventilated with a V_T_ of 7 mL x kg^-1^ compared with animals ventilated with a V_T_ of 15 mL x kg^-1^ [29]. In this study, the increased nitrite levels in urine in the protectively ventilated animals support the results of Vobruba et al. and serve as a mechanistic explanation to induction of endotoxin tolerance following protective ventilation. Furthermore, the association between higher levels of endotoxin tolerance, elevated urinary nitrite and lower blood pressure seen in one of our recent investigations supports the findings of the present study [15].

It has been questioned whether the compensatory rise in respiratory rate following low V_T_ ventilation can lead to injurious effects [30]. Higher respiratory rate in spontaneously breathing rats led to increased activation of lung tissue metalloproteinases and reduced integrity of the extracellular matrix in the lung [31]. Wang et al. reported an association between metalloproteinase 9 and upregulation of iNOS via nuclear factor-κβ [32]. It might be hypothesized that the higher respiratory rate in low V_T_ ventilation leads to effects on the lung extracellular matrix, which, in turn, induces iNOS and endotoxin tolerance. 

What the present study adds is the beneficial effect of preventive treatment with low V_T_ ventilation 2 h before an experimental septic insult. The inflammatory response to standardized surgery together with mechanical ventilation did not differ between the groups at 0 h (Figures 2,3,4). Similarly, the pulmonary function and the other organ function variables were the same in all groups at 0 h. Despite this observation, there were obvious inflammatory differences between the groups induced by the simulated postoperative complication, i.e. the endotoxin infusion, which indicates that one main effect of different perioperative ventilator protocols may be induction of immunotolerance after low V_T_ ventilation. This assumption is reinforced with the endotoxin tolerant feature of the protectively ventilated animals associated with higher urinary nitrite levels.

This study was conducted in a large animal model, which is a strength compared with previous investigations conducted in small animal models. The porcine anatomy and physiology have many similarities with humans and the size of the animal facilitates the use of invasive catheters, fluid protocols and circulatory interventions that approximate those in clinical patient use [33,34]. 

The fact that the animals that died before the experimental end point was replaced with new animals raises a methodological problem mainly due to a possible selection of fitter animals. To not replace them would have presented other problems such as not having enough statistical power, or the problem of multiple missing values. To assess the impact of the replaced animals, the statistical analyses were also conducted without the replacement animals in group Prot-5h, a strategy that did not change the results of the study. 

It has been argued that endotoxin as a model of sepsis is insufficient to produce the complete pathophysiological changes noted in clinical sepsis [35]. On the other hand, endotoxin induces a predictable systemic inflammation [17], which served well to answer the proposed aims of this study. A limitation of greater importance is the short observation period of this study. The differences of borderline statistical significance noted in this study could be attributed to low statistical power, since the power calculations were based on the primary outcome variables, i.e. inflammatory mediators. In this model, the inflammatory response peaks already one to three hours after the start an endotoxin infusion, whereas the development of organ dysfunction is usually noted towards the end of a six-hour endotoxin infusion experiment [36]. It therefore seems likely that some of the variables that displayed borderline differences would exhibit significant differences during an extended experimental period. It would also be of interest to repeat the present study in iNOS knockout animals or with concurrent administration of an iNOS antagonist, e.g., aminoguanidine, to study whether the beneficial effects of protective ventilation could be reduced or even eliminated. 

## Conclusions

Low V_T_ ventilation combined with higher PEEP in healthy animals exposed to surgery and experimental postoperative sepsis led to a less prominent systemic inflammatory response, pulmonary dysfunction and cardiac injury when compared with animals ventilated with medium-high V_T_ and lower PEEP. Preventive low V_T_ ventilation was associated with immunotolerance and higher nitric oxide production, which could be a mechanistic explanation for the attenuation of systemic inflammation. 
